# Impact of Body Mass Index on Short-Term Outcomes in Patients Undergoing Percutaneous Coronary Intervention in Newfoundland and Labrador, Canada

**DOI:** 10.1155/2016/7154267

**Published:** 2016-09-07

**Authors:** Anne B. Gregory, Kendra K. Lester, Deborah M. Gregory, Laurie K. Twells, William K. Midodzi, Neil J. Pearce

**Affiliations:** ^1^Eastern Health, St. John's, NL, Canada A1B 3V6; ^2^Department of Clinical Epidemiology, Faculty of Medicine, Memorial University of Newfoundland, St. John's, NL, Canada A1B 3V6; ^3^Department of Medicine, Faculty of Medicine, Memorial University of Newfoundland, St. John's, NL, Canada A1B 3V6; ^4^School of Pharmacy, Memorial University of Newfoundland, St. John's, NL, Canada A1B 3V6

## Abstract

*Background and Aim*. Obesity (BMI ≥ 30 kg/m^2^) is associated with advanced cardiovascular disease requiring procedures such as percutaneous coronary intervention (PCI). Studies report better outcomes in obese patients having these procedures but results are conflicting or inconsistent. Newfoundland and Labrador (NL) has the highest rate of obesity in Canada. The aim of the study was to examine the relationship between BMI and vascular and nonvascular complications in patients undergoing PCI in NL.* Methods*. We studied 6473 patients identified in the APPROACH-NL database who underwent PCI from May 2006 to December 2013. BMI categories included normal, 18.5 ≤ BMI < 25.0 (*n* = 1073); overweight, 25.0 ≤ BMI < 30 (*n* = 2608); and obese, BMI ≥ 30.0 (*n* = 2792).* Results*. Patients with obesity were younger and had a higher incidence of diabetes, hypertension, and family history of cardiac disease. Obese patients experienced less vascular complications (normal, overweight, and obese: 8.2%, 7.2%, and 5.3%, *p* = 0.001). No significant differences were observed for in-lab (4.0%, 3.3%, and 3.1%, *p* = 0.386) or postprocedural (1.0%, 0.8%, and 0.9%, *p* = 0.725) nonvascular complications. After adjusting for covariates, BMI was not a significant factor associated with adverse outcomes.* Conclusion*. Overweight and obesity were not independent correlates of short-term vascular and nonvascular complications among patients undergoing PCI.

## 1. Introduction

Obesity is an independent risk factor for cardiovascular disease [1–5] and is associated with advanced cardiovascular disease requiring procedures such as percutaneous coronary intervention (PCI) and coronary artery bypass grafting (CABG), reduction in life expectancy [6], and a higher mortality rate [3, 7, 8]. A number of observational studies have reported improved clinical outcomes (i.e., increased survival benefit) in overweight and obese patients treated for cardiovascular diseases compared to normal weight patients, a phenomenon commonly referred to as “obesity paradox” [9–14]. This phenomenon is considered to be counterintuitive, referred to as “reverse epidemiology,” and reported in patients with hypertension [15], heart failure [16], coronary artery disease (CAD) [15, 17–19], CABG [17–20], and PCI [17–19]. The term “reverse epidemiology” was first used in a 2003 study by Kalantar-Zadeh et al. [21] which focused on cardiovascular risk factors including body mass index (BMI), serum cholesterol, and blood pressure in maintenance hemodialysis patients. Obesity, hypercholesterolemia, and hypertension were paradoxically associated with survival, the opposite of that observed in the general population. In 2004, Kalantar-Zadeh et al. also reported a protective role of conventional cardiovascular risk factors in chronic heart failure [22]. Use of the term “reverse epidemiology” and alternative terms including “risk factor reversal” and “altered risk factor patterns” [23] has continued in the research literature; however, some refer to the term as confusing, confounding, and inaccurate [24] and suggest it may be a “questionable concept.” Inconsistent results have been reported regarding the association between BMI and short-term clinical outcomes (i.e., vascular complication, nonvascular in-lab and postprocedural complications) and/or mortality in patients undergoing PCI [9, 10, 13, 25–31]; therefore, it is not entirely clear whether an obesity paradox exists.

Obesity is a common and rapidly growing public health concern. Between 1985 and 2011, the prevalence of this disease in Canada increased by 200% from 6.1% to 18.3% equating to more than 4.8 million adults, with continued increases projected [32]. Newfoundland and Labrador (NL) has the highest rate of obesity in Canada. It is estimated that 71% of the province's population will be either overweight or obese by 2019 [32]. There is a paucity of data on the prevalence of obesity in patients undergoing PCI in the province. Furthermore, the relationship between short-term clinical outcomes and BMI has not been examined in patients undergoing PCI in NL. In the present study, we (1) examine the prevalence of obesity among patients undergoing PCI and the differences among BMI groups on demographic, clinical, and procedural findings and (2) examine the association between the most commonly used anthropometric parameter to assess adiposity (i.e., BMI) and short-term outcomes (vascular complication, nonvascular in-lab and postprocedural complications occurring within 48 hours).

## 2. Methods

### 2.1. Study Design

We performed a retrospective analysis of prospectively collected deidentified data for all patients 18 years of age and older who had a PCI between May 1, 2006, and December 31, 2013, in the province of NL, Canada, using a well-established clinical database (i.e., Alberta Provincial Project for Outcome Assessment in Coronary Heart Disease-Newfoundland and Labrador (APPROACH-NL)). Detailed prospective demographic, clinical, and procedural data on all patients undergoing diagnostic cardiac catheterization and/or percutaneous coronary intervention (PCI) and cardiac surgery since 2006 is collected by specifically trained clinical cardiac catheterization database nurses. Nurses collect and record, on an abstraction sheet, patient data provided by nurses responsible for the care of the patient which includes examination and assessment of the access site for potential vascular complications. The attending physician also examined the vascular access site. All data are verified by chart review until hospital discharge by these nurses. Prospectively collected data on each consecutive patient is entered into the APPROACH-NL clinical database. A research nurse is responsible for the management of the database including completeness of data entry and quality assurance activities. Details of the database and methods of collection have been previously described [33]. If patients are not hospitalized, they remain in the local area for 24 hours and are advised to return to the emergency department (ER) if they encounter any problems. ER admissions are audited by a clerk in the cardiac catheterization laboratory in the event that a patient returns to the ER.

### 2.2. Study Population

For the current study, all consecutive PCIs (*N* = 6633) performed on patients 18 years of age and older between May 1, 2006, and December 31, 2013, at the Health Science Centre, Eastern Health, NL, were enrolled. PCI procedures performed on underweight (BMI < 18.5 kg/m^2^) individuals (*n* = 47) or those with missing BMI data or unlikely valid BMI levels of >70 or <11 kg/m^2^ (*n* = 113) were excluded. The remaining patients comprised the study cohort. Based on these selection criteria, 6,473 patients were included in the final analysis.

Weight and height were measured and documented by a nurse at the time of PCI. If patients were unstable, self-reported weight and height were collected and BMI was calculated. Patients were grouped according to three BMI categories using the World Health Organization classification system: normal (18.5–24.9 kg/m^2^), overweight (25.0–29.9 kg/m^2^), and obese (≥30 kg/m^2^) [34]. These categorizations reflect relative increasing levels of risk to health [35].

### 2.3. Clinical Outcomes and Definitions

The* primary outcome* was short-term complications occurring within 48 hours after the intervention. The clinical definitions for complications were as follows.* Vascular access complications *were defined as hematoma (>5 cm), pseudoaneurysm, arteriovenous fistula, vascular occlusion, access site bleeding, retroperitoneal bleed, loss of distal pulse, or occlusion.* Nonvascular complications *included in-lab events (abrupt coronary closure, emergency CABG, access site complications, death, ventricular tachycardia/ventricular fibrillation, pulmonary edema, shock, and dissection) and* postprocedural complications* included death, myocardial infarction, emergency CABG, abrupt coronary closure, hemorrhagic or ischemic CVA, and GI bleed. Each of the outcomes was a composite of the individual outcomes defined in each category.

### 2.4. Ethical Considerations

All patients who had a PCI during the time period under examination gave written informed consent to the cardiac care program for data collection and follow-up observation after PCI. The study protocol was approved by the Health Research Ethics Authority of Memorial University and Eastern Health.

### 2.5. Data Analysis

Demographic characteristics and clinical and procedural related variables were summarized. Continuous variables were expressed as mean ± standard deviation (SD). Categorical variables were expressed as frequencies and percentages. Continuous variables were compared using ANOVA, and the differences between categorical variables were examined using the *χ* ^2^ test and, where appropriate, the Fisher exact test is reported. All *p* values were two-tailed, with statistical significance defined by a *p* value < 0.05. Comparisons were performed for a trend in increasing BMI categories using *χ* ^2^ test for trends. Univariate logistic regression analysis was performed to determine the odds ratio for vascular complications and nonvascular complications occurring in the cardiac care laboratory identified within 24–48 hours after PCI. Multivariate logistic regression analysis was used to examine independent predictors for each of the patient outcomes. Due to the low nonvascular postprocedural complication event rate, regression analyses were not performed. Variables identified in Tables 1 [Table tab2]–3 were selected for these models based on univariate *p* values < 0.20 and overall clinical significance. All statistical analyses were performed using IBM SPSS Statistics for Windows, Version 22.0 (IBM Corp., Armonk, NY) [36].

## 3. Results

**Figure 1:**
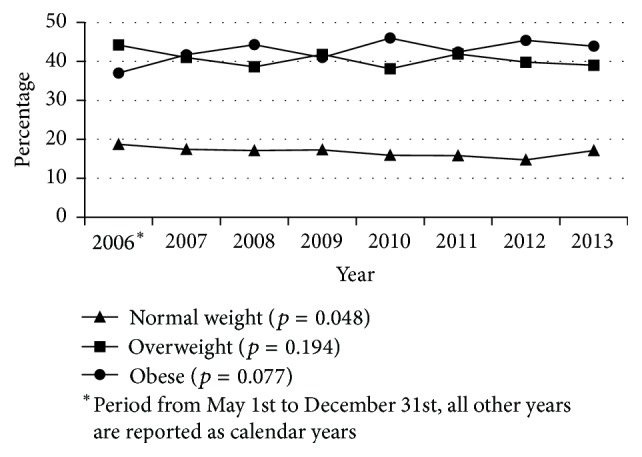
Body mass index trends for normal weight, overweight, and obese patients from 2006 to 2013.

A cohort of 6,473 patients was identified from the population of patients who had a PCI during the time period under examination. BMI values for normal weight, overweight, and obese patients from 2006 to 2013 are presented in Figure 1. Tables 1 [Table tab2]–3 show the baseline characteristics of patients according to categories of BMI, medications at time of referral, and admitting clinical angiographic and procedural data.

### 3.1. Baseline Characteristics

Of the 6,473 patients, 16.6% were of normal weight (*n* = 1073), 40.3% were overweight (*n* = 2608), and 43.1% were obese (*n* = 2792). In each of the years examined, less than 19% of patients who had a PCI were of normal weight (Table 1 and Figure 1). The baseline characteristics of the study patients according to the three BMI categories are presented in Table 1. There were statistically significant differences between the groups on a number of characteristics. A higher proportion of overweight patients were male. Patients with obesity were younger, had a higher incidence of coronary risk factors such as diabetes mellitus and hypertension, and had a family history of coronary artery disease. Patients with a higher BMI were also more likely to have COPD, whereas normal weight patients were more likely to have PVD. No significant differences were observed in smoking status.

Medications at the time of referral for PCI were examined. The details regarding the use of medications prior to PCI are presented in Table 2. No significant differences were found in the use of acetylsalicylic acid, coumadin, preprocedural GP IIb/IIIa inhibitors, beta blockers, LMWH, IV heparin, IV nitrates, or statin therapy between the groups. Patients with obesity were less likely to receive the antiplatelet medication ticlopidine/clopidogrel but were more likely to receive an angiotensin-converting enzyme inhibitor/angiotensin receptor blocker, calcium channel blockers, and long-acting nitrates.

### 3.2. Angiographic and Procedural Data

Admitting clinical, angiographic, and procedural data are shown in Table 3. Normal weight patients were significantly less likely to require a closure device (*p* < 0.001) compared to other BMI groups. However, there were no significant differences among the BMI categories in the prevalence of prior PCI, prior CABG, prior HF, prior MI, pulmonary embolism, thromboembolic history, deep vein thrombosis, same sitting angioplasty, IABP use at time of referral or during the procedure/cardiogenic shock at time of procedure, use of GP IIb/IIIa inhibitors, access site, and choice of sheath size. A greater proportion of normal weight patients presented as emergency/urgent cases, whereas more elective procedures were performed in overweight and obese patients. A greater proportion of obese patients presented with unstable angina, whereas a much lower proportion presented with a STEMI.

### 3.3. Complications Occurring within 24 to 48 Hours of PCI according to BMI

**Figure 2:**
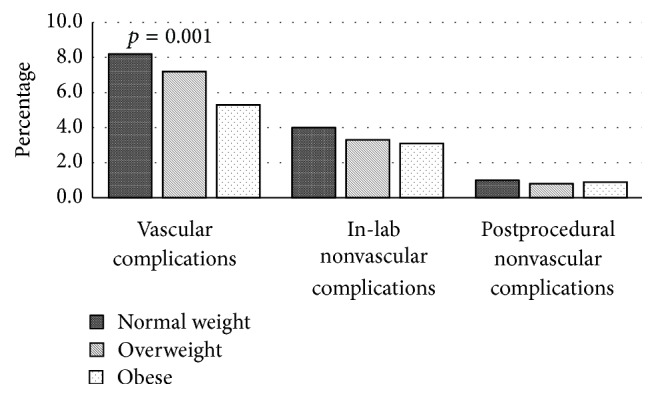
Prevalence of vascular and nonvascular complications (in-lab and postprocedural) by body mass index category.

Complications occurring within 24 to 48 hours of PCI according to BMI category are presented in Table 4 and Figure 2. Obese subjects experienced a lower proportion of vascular complications (normal, overweight, and obese: 8.2%, 7.2%, and 5.3%, *p* = 0.001). No significant differences were observed for nonvascular complications either in-lab (4.0%, 3.3%, and 3.1%, *p* = 0.386) or postprocedural (1.0%, 0.8%, and 0.9%, *p* = 0.725).

We performed multivariate analyses to adjust for clinical and procedural characteristics. Independent factors associated with the primary outcomes of vascular complications and nonvascular in-lab complications are shown in Tables 5 and 6. Increasing age, GP IIb/IIIa inhibitor, and LMWH use during the procedure and the utilization of a femoral access site approach were significant factors associated with the occurrence of vascular complications. Males, patients with diabetes and patients who had a closure device, and PCIs performed in 2010 were less likely to have vascular complications. GI/liver disease, coumadin use, utilization of GP IIb/IIIa inhibitors or IV heparin during PCI, and older age were significant factors associated with the occurrence of nonvascular in-lab complications. Male sex and the use of a closure device were protective factors associated with a less likelihood of nonvascular in-lab complications. BMI was not a significant factor associated with either vascular or nonvascular in-lab complications (Tables 5 and 6).

## 4. Discussion

The present study examined all adult patients who had a PCI procedure performed between 2006 and 2013 in one Canadian province to determine the prevalence of obesity in this patient population and trend in rates over time. A second objective was to examine the relationship between BMI and short-term vascular and nonvascular complications occurring within 48 hours and compare outcomes among three BMI categories (normal weight, overweight, and obese). The majority of patients (84.3%) were either overweight or obese. Our study findings are comparable to other studies that have used PCI registries [10, 11, 30]. In the current study, we found that over time there was a significant trend of decreasing prevalence for the normal weight category of patients undergoing PCI (*p* = 0.048). Similar to previous studies, the current study demonstrates that obese patients presented with more risk factors for CAD than overweight or normal weight patients. Obese patients were younger, diabetic, and hypertensive and had higher rates of hyperlipidemia and family history of CAD.

We hypothesized that BMI was an independent correlate of outcome in patients undergoing PCI; more specifically, obese patients would experience worse outcomes compared to normal and overweight patients. The obese patients in the present study were significantly younger and had higher incidence of coronary risk factors such as diabetes mellitus and hypertension and had a family history of coronary artery but based on the findings of the univariate analyses had a significantly lower rate of vascular complications (hematoma (>5 cm), pseudoaneurysm, arteriovenous fistula, vascular occlusion, access site bleeding, retroperitoneal bleed, loss of distal pulse, or occlusion) than their normal weight and overweight counterparts. There were no significant differences in the rates of nonvascular in-lab (acute coronary closure, emergency CABG, access site complications, death, ventricular tachycardia/ventricular fibrillation, pulmonary edema, shock, and dissection) and postprocedural complications (death, myocardial infarction, emergency CABG, abrupt coronary closure, hemorrhagic or ischemic CVA, and GI bleed) among the BMI categories.

After multiple logistic regression analysis, BMI was not a significant predictor of short-term outcomes (vascular complications or in-lab nonvascular complications). Our data regarding BMI in NL patients is consistent with one previous Canadian study but is contradictory to the findings of a 2009 study conducted by Byrne et al. [28]. Similar to our findings, Shubair et al. [10] evaluated the effect of BMI on in-hospital outcomes in a consecutive series of coronary artery disease patients undergoing PCI enrolled in a clinical database at the Hamilton Health Sciences in Ontario, Canada. The authors found that obesity was not associated with in-hospital postprocedural death, myocardial infarction, repeat PCI, CABG, or major adverse cardiac event defined as a composite of death, myocardial infarction, repeat PCI, and CABG. Using a large Canadian provincial registry, Byrne et al. [28] investigated the relationship between BMI, bleeding, and outcome (i.e., 1-year mortality) after PCI. The authors reported that lower BMI (≤18.5 kg/m^2^) and higher BMI (≥40 kg/m^2^) patients were at greater risk of bleeding and death after PCI. Other studies conducted in the western society have reported that underweight [9, 25, 28], normal weight [9, 25], and extremely obese [29–31] patients are at greater risk of adverse outcomes after PCI. Cox et al. [9] reported that the rate of vascular complications was the highest in extremely thin and morbidly obese patients and the lowest in moderately obese patients. In a study by Gruberg et al. [25], the authors reported that normal weight patients were at the highest risk of in-hospital complications (i.e., major bleeding, vascular complications, emergency CABG, and myocardial infarction) and cardiac death compared to overweight and obese patients. Two studies by Gurm et al. [26, 27] suggested that being moderately obese conferred a protective effect, referred to as an “obesity paradox,” in relation to vascular complications and major adverse outcomes after PCI, a finding consistent with that reported by Cox et al. [9].

In other studies that have focused primarily on the comparison of normal weight and extremely obese (≥40 kg/m^2^) patients undergoing PCI, researchers have reported that extremely obese patients have increased vascular complications [30] compared to normal weight individuals and higher rates of in-hospital mortality [29–31] compared to overweight individuals. In the current study, we were unable to examine the various classes of obesity due to the small numbers in each category.

*Study Strengths and Limitations*. Our study has a number of strengths. We report on a large population-based cohort of patients undergoing PCI at a single tertiary cardiac centre using APPROACH-NL prospectively collected data. Data quality assurance indicated that the amount of missing data was minimal (1.7%). Actual measures of height and weight were taken at the time of the procedure unless the patients were unstable.

This study also has a number of limitations. Our study is an observational nonrandomized cohort study with retrospective analysis. The current study design can only establish association and not causation. We used data from a clinical database and as such cannot account for confounders not captured in the database. The study population was heterogeneous (i.e., included patients with variable levels of coronary artery disease severity ranging from acute coronary syndrome with cardiogenic shock to stable angina). Patients with missing BMI data were excluded (*n* = 113) which may contribute to selection bias, but as missing data only accounted for 1.7% this is unlikely. Despite its widespread use, the use of BMI in terms of its accuracy to define obesity is controversial [37–39]. BMI is not as well correlated to cardiovascular disease and death as other measures including waist circumference and waist-to-hip ratio [40], data that were unavailable in the clinical database. A lack of underweight and severely obese patients meant that comparisons in our study were made between only three BMI groups: normal weight, overweight, and obese.

## 5. Conclusion

Overweight and obesity were not independent predictors of short-term outcomes (vascular or nonvascular complications occurring within 24 to 48 hours) in patients undergoing PCI at our institution.

## Figures and Tables

**Table 1:** Baseline characteristics of patients according to categories of BMI.

Variable	Total *N*	NW	OW	OB	*p* value^*∗*^
Number of patients	6473	1073	2608	2792	

Age, years	6473	65.1 ± 11.1	63.1 ± 10.5	60.7 ± 10.1	*p* < 0.001

Male sex	6473	695 (64.8)	1975 (75.7)	1945 (69.7)	*p* < 0.001

Cardiovascular risk factors					
HTN	6462	658 (61.4)	1661 (63.8)	2066 (74.1)	*p* < 0.001
Hyperlipidemia	6462	905 (84.4)	2241 (86.1)	2434 (87.4)	*p* = 0.050
Diabetes	6464	226 (21.1)	637 (24.5)	1040 (37.3)	*p* < 0.001
Family history	6440	622 (58.3)	1627 (62.7)	1822 (65.5)	*p* < 0.001

Smoking status	6421				
Never	1719	298 (28.1)	698 (27.0)	723 (26.1)	*p* = 0.441
Smoking history	4702	763 (71.9)	1891 (73.0)	2048 (73.9)	

PVD	6460	91 (8.5)	172 (6.6)	156 (5.6)	*p* = 0.005

COPD	6459	156 (14.6)	335 (12.9)	479 (17.2)	*p* < 0.001

Values are presented as *n* (%) or mean ± SD, as indicated.

^*∗*^ *p* values for chi-squared or ANOVA tests.

BMI: body mass index; COPD: chronic obstructive pulmonary disease; NW: normal weight; OB: obese; OW: overweight; HTN: hypertension; PVD: peripheral vascular disease.

**Table 2 tab2:** Medications at time of referral for PCI by BMI category.

	Total *N*	NW	OW	OB	*p* value^*∗*^
Number of patients	6473	1073	2608	2792	
Beta blockers	6431	863 (81.2)	2154 (83.1)	2318 (83.5)	*p* = 0.220
ACE inhibitors	6429	510 (48.0)	1296 (50.0)	1476 (53.2)	*p* = 0.006
ARB antagonist	6428	104 (9.8)	305 (11.8)	431 (15.5)	*p* < 0.001
CCB	6430	173 (16.3)	433 (16.7)	596 (21.5)	*p* < 0.001
LA nitrates	6430	307 (28.9)	729 (28.1)	869 (31.3)	*p* = 0.032
Statin therapy	6427	874 (82.3)	2199 (84.9)	2345 (84.5)	*p* = 0.134
Aspirin	6432	983 (92.5)	2402 (92.6)	2612 (94.1)	*p* = 0.058
Ticlopidine/clopidogrel	6432	806 (75.8)	1846 (71.2)	1925 (69.3)	*p* < 0.001
Coumadin	6429	15 (1.4)	47 (1.8)	59 (2.1)	*p* = 0.329
GP IIb/IIIa inhibitors	6437	5 (0.5)	14 (0.5)	13 (0.5)	*p* = 0.924
LMWH	6439	428 (40.2)	971 (37.4)	1003 (36.1)	*p* = 0.068
IV heparin	6439	220 (20.6)	497 (19.1)	579 (20.8)	*p* = 0.268
IV nitrates	6430	141 (13.3)	313 (12.1)	307 (11.1)	*p* = 0.149

Values are presented as *n* (%).

^*∗*^ *p* values for chi-squared tests.

ACE: angiotensin converting enzyme; ARB: angiotensin receptor blocker; BMI: body mass index; CCB: calcium channel blockers; LA nitrates: long-acting nitrates; LMWH: low molecular weight heparin; NW: normal weight; OB: obese; OW: overweight; PCI: percutaneous coronary intervention.

**Table 3:** Admitting clinical, angiographic, and procedural data for patients undergoing PCI according to BMI category.

	Total *N*	NW	OW	OB	*p* value^*∗*^
Number of patients	6473	*n* = 1073	*n* = 2608	*n* = 2792	

Cardiovascular history					
Prior PCI	6462	226 (21.1)	543 (20.9)	627 (22.5)	*p* = 0.318
Prior CABG	6462	129 (12.0)	298 (11.5)	289 (10.4)	*p* = 0.247
Prior HF	6462	52 (4.9)	83 (3.2)	106 (3.8)	*p* = 0.052
Prior MI	6462	212 (19.8)	527 (20.3)	581 (20.8)	*p* = 0.734
CVD	6450	89 (8.3)	160 (6.2)	174 (6.3)	*p* = 0.041

Same sitting angioplasty	6473	864 (80.5)	2138 (82.0)	2321 (83.1)	*p* = 0.149

IABP/cardiogenic shock	6461	7 (0.7)	16 (0.6)	31 (1.1)	*p* = 0.104

Priority	6466				
Low risk	1861	220 (20.5)	783 (30.0)	858 (30.7)	*p* < 0.001
Emergency	397	72 (6.7)	170 (6.5)	155 (5.6)	
Urgent	4208	780 (72.7)	1654 (63.4)	1774 (63.5)	

PE	6216	8 (0.8)	13 (0.5)	15 (0.6)	*p* = 0.641

Thromboembolic history	6218	5 (0.5)	12 (0.5)	10 (0.4)	*p* = 0.797

DVT	6219	16 (1.6)	32 (1.3)	32 (1.2)	*p* = 0.663

Stable angina	1748	202 (18.9)	731 (28.1)	815 (29.3)	*p* < 0.001

ACS	4341	*n* = 791	*n* = 1724	*n* = 1825	
STEMI	1227	251 (31.7)	501 (29.1)	475 (26.0)	*p* = 0.001
Non-STEMI	1939	349 (44.1)	787 (45.6)	803 (44.0)	
Unstable angina	1174	191 (24.1)	436 (25.3)	547 (30.0)	
Thrombolytics contraindicated	4143	11 (1.5)	27 (1.6)	24 (1.4)	*p* = 0.831
Failed thrombolysis	4274	26 (3.4)	77 (4.5)	59 (3.3)	*p* = 0.122

Access site					
Radial/brachial	1172	193 (18.0)	468 (17.9)	511 (18.3)	*p* = 0.938
Femoral	5301	880 (82.0)	2140 (82.1)	2282 (81.7)	

Sheath size					
Sheath size 5 Fr	924	160 (14.9)	393 (15.1)	371 (13.3)	*p* = 0.386
Sheath size 6 Fr	5432	893 (83.2)	2171 (83.3)	2368 (84.9)	
Sheath size 7/8 Fr	114	20 (1.9)	43 (1.6)	51 (1.8)	

Closure device	6470	484 (45.1)	1359 (52.1)	1506 (54.0)	*p* < 0.001

GP IIb/IIIa inhibitors	6471	134 (12.5)	338 (13.0)	351 (12.6)	*p* = 0.889

Values are presented as *n* (%).

^*∗*^ *p* values for chi-squared tests.

ACS = acute coronary syndrome; BMI: body mass index; CABG: coronary artery bypass grafting; CVD: cerebrovascular disease; DVT= deep vein thrombosis; HF: heart failure; IABP: intra-aortic balloon pump; MI: myocardial infarction; NW: normal weight; OB: obese; OW: overweight; PCI: percutaneous coronary intervention; PE: pulmonary embolism; PVD: peripheral vascular disease; STEMI: ST elevation myocardial infarction.

**Table 4:** Vascular and nonvascular complications occurring within 24 to 48 hours in patients undergoing PCI according to BMI category.

	NW (*n* = 1073)	OW (*n* = 2608)	OB (*n* = 2792)	*p* value^*∗*^
Vascular complications	88 (8.2)	187 (7.2)	149 (5.3)	0.001
Nonvascular in-lab complications	43 (4.0)	87 (3.3)	87 (3.1)	0.386
Nonvascular postprocedural complications	11 (1.0)	20 (0.8)	25 (0.9)	0.725

Values are presented as *n* (%).

BMI: body mass index; NW: normal weight; OW: overweight; OB: obese; PCI: percutaneous coronary intervention.

^*∗*^ *p* values for chi-squared tests.

Vascular complications were defined as hematoma (>5 cm), pseudoaneurysm, arteriovenous fistula, vascular occlusion, access site bleeding, retroperitoneal bleed, loss of distal pulse, or occlusion.

Nonvascular complications occurring in-lab included abrupt coronary closure, emergency coronary artery bypass grafting (CABG), access site complications, death, ventricular tachycardia/ventricular fibrillation, pulmonary edema, shock, and dissection.

Nonvascular postprocedural complications included death, myocardial infarction, emergency CABG, abrupt coronary closure, hemorrhagic or ischemic CVA, and GI bleed.

**Table 5:** Multivariate adjusted OR for vascular complications in patients undergoing PCI.

	OR	95% CI	*p* value
Age	1.02	1.01–1.03	0.001
Male	0.69	0.55–0.86	0.001
Diabetes	0.65	0.51–0.84	0.001
Sheath size 5 Fr	0.42	0.20–0.85	0.016
Procedural GP IIb/IIIa inhibitors	1.95	1.50–2.54	0.000
Preprocedural LMWH	1.29	1.03–1.61	0.029
Closure device	0.54	0.43–0.68	0.000
Femoral access	2.98	2.00–4.45	0.000
Year, 2010	0.49	0.30–0.80	0.005
BMI (referent category is normal weight)			
Overweight	1.01	0.76–1.33	0.967
Obese	0.83	0.62–1.11	0.219

Adjusted for access site, age, aspirin, BMI, closure device, diabetes, gender, GI/liver disease, LMWH in-lab, preprocedural LMWH, GP IIb/IIIa inhibitors in-lab, preprocedural GP IIb/IIIa inhibitors, preprocedural IV heparin, prior CVD, prior HF, prior PCI, sheath size, smoking status, ticlopidine/clopidogrel, and year.

BMI: body mass index; CVD: cardiovascular disease; GP: glycoprotein; HF: heart failure; LMWH: low molecular weight heparin; PCI: percutaneous coronary intervention.

**Table 6:** Multivariate adjusted OR for nonvascular in-lab complications in patients undergoing PCI.

	OR	95% CI	*p* value
Age	1.02	1.001–1.03	0.039
Male	0.64	0.47–0.87	0.005
GI/liver disease	1.53	1.02–2.27	0.038
GP IIb/IIIa inhibitors in-lab	4.99	3.65–6.81	0.000
IV heparin in-lab	1.70	1.14–2.52	0.009
Coumadin	2.38	1.14–4.98	0.021
Closure device	0.28	0.19–0.40	0.000
BMI (referent category is normal weight)			
Overweight	0.93	0.62–1.38	0.705
Obese	0.54	0.58–1.32	0.879

Adjusted for age, BMI, closure device, coumadin, diabetes, DVT, family history of premature CAD, GP IIb/IIIa inhibitors in-lab, gender, GI/liver disease, hypertension, IV heparin in-lab, LMWH in-lab, prior CABG, prior COPD, prior CVD, prior HF, prior PCI, prior PVD, pulmonary embolism, sheath size, and year.

BMI: body mass index; CAD: coronary artery disease; CABG: coronary artery bypass grafting; COPD: chronic obstructive pulmonary disease; CVD: cardiovascular disease; DVT: deep vein thrombosis; GP: glycoprotein; HF: heart failure; LMWH: low molecular weight heparin; PCI: percutaneous coronary intervention; PVD: peripheral vascular disease.
